# Impact of a POCUS-first versus CT-first approach on emergency department length of stay and time to surgical consultation in patients with acute cholecystitis: a retrospective study

**DOI:** 10.1186/s13049-025-01341-2

**Published:** 2025-02-10

**Authors:** Chien-Tai Huang, Liang-Wei Wang, Shao-Yung Lin, Tai-Yuan Chen, Yi-Ju Ho, Pei-Hsiu Wang, Kao-Lang Liu, Yao-Ming Wu, Hsiu-Po Wang, Wan-Ching Lien

**Affiliations:** 1 Department of Emergency Medicine, National Taiwan University Hospital Hsin-Chu Branch, Hsinchu City, Taiwan; 2 Department of Emergency Medicine, College of Medicine, National Taiwan University, Taipei, Taiwan; 3 Department of Emergency Medicine, National Taiwan University Hospital, No. 7, Chung-Shan South Road, Taipei, 100 Taiwan; 4 Department of Emergency Medicine, National Taiwan University Hospital Yun-Lin Branch, Douliu City, Taiwan; 5 Section of Emergency Medicine, Department of Medicine, National Taiwan University Cancer Center, National Taiwan University Hospital, Taipei, Taiwan; 6 Department of Medical Imaging, National Taiwan University Cancer Center, National Taiwan University Hospital, Taipei, Taiwan; 7 Department of Medical Imaging, College of Medicine, National Taiwan University, Taipei, Taiwan; 8 Department of Surgery, College of Medicine, National Taiwan University, Taipei, Taiwan; 9 Department of Internal Medicine, College of Medicine, National Taiwan University, Taipei, Taiwan

**Keywords:** Point-of-care ultrasound, Computed tomography, Emergency department, Acute cholecystitis, Length of stay, Surgical consultation

## Abstract

**Objective:**

This study aims to evaluate the impact of point-of-care ultrasound (PoCUS) and computed tomography (CT) on emergency department (ED) length of stay (LOS) and time to surgical consultation in patients with mild acute cholecystitis (AC).

**Methods:**

Adult patients with CT-confirmed grade I AC were retrospectively enrolled and divided into the PoCUS-first group and the CT-first group. The primary outcome was the relationship between the door-to-ultrasound (US)/CT time and ED-LOS. The secondary outcome was the relationship between the door-to-US/CT time and time to surgical consultation.

**Results:**

A total of 1627 patients were included with 264 in the PoCUS first group. In the PoCUS group, door-to-US time was positively associated with ED-LOS (β = 0.27, *p* < 0.001) and time to surgical consultation (β = 0.36, *p* < 0.001). Similarly, door-to-CT time was also positively associated with ED-LOS (β = 0.21, *p* < 0.001) and time to surgical consultation (β = 0.75, *p* < 0.001) in the CT group. Conducting PoCUS within 60 min was associated with a reduced ED-LOS and time to surgical consultation, resulting in a saving of 22.4 h and 266 min, respectively. In the CT group, performing CT within 120 min was associated with a reduced ED-LOS and time to surgical consultation, resulting in a decrease of 12 h and 188 min, respectively. The ED-LOS and time to surgical consultation were similar between patients receiving PoCUS within 60 min in PoCUS group and those receiving CT within 120 min in the CT group.

**Conclusions:**

Performing PoCUS within 60 min or CT within 120 min was associated with shorter ED-LOS and earlier surgical consultation, enhancing the ED efficiency in patients with mild AC.

*Trial registration***:** NCT04149041 at ClinicalTrial.gov.

**Supplementary Information:**

The online version contains supplementary material available at 10.1186/s13049-025-01341-2.

## Introduction

Acute cholecystitis (AC) is a common condition in the emergency department (ED), accounting for 3–10% of patients presenting with acute abdominal pain [1]. The diagnosis of AC is based on a combination of clinical presentation, laboratory findings, and imaging results, as outlined by the Tokyo guidelines [2]. Ultrasound (US) is recommended as the initial imaging modality due to its cost-effectiveness, wide availability, and non-invasive nature [2, 3]. However, computed tomography (CT) is also frequently used in the ED, as it can provide additional information to guide further management, including emergent or delayed cholecystectomy [4].

Point-of-care ultrasound (PoCUS), performed by emergency physicians, has been shown to have diagnostic accuracy comparable to radiologist-performed US in identifying AC [5]. However, evidence regarding the impact of PoCUS and CT on patient-centered outcomes, such as ED length of stay (LOS) and the management process, remains limited in patients with AC [6–9].

Previous studies have reported that the interval from ED presentation to operative intervention for AC typically ranges from 10 to 34 h [10–12]. Hospital stays of 3–7 days are common among patients managed non-operatively, longer than those receiving emergency cholecystectomy [1, 13, 14]. However, it remains unclear whether PoCUS can streamline ED management processes, such as reducing ED-LOS and time to surgical consultation, thereby potentially mitigating ED crowding.

This study aims to compare ED-LOS and time to surgical consultation in ED patients with mild AC undergoing a POCUS-first versus CT-first approach.

## Materials and methods

### Study design and setting

The retrospective study was conducted at the ED of the National Taiwan University Hospital (NTUH), an academic medical center, from July 2012 to June 2020. The ED serves an annual average of 100,000 patient visits, with ED admissions comprising only 18% of the total hospital admissions. The study was approved by the Institutional Review Board of the Ethics Committee of the NTUH (201907173RIND) with a waiver of informed consent and registered at ClinicalTrials.gov (NCT04149041).

Two US machines (SSA-550A, SSA-660, Canon, Japan) equipped with 2–5 MHz curvilinear transducers were set up and placed on standby for use.

### Study population

Eligible adult patients (more than 18 years) with CT-confirmed AC were identified by electronic medical records. In our hospital, CT is performed in all patients with AC. Patients with duplicate data or grade II-III AC according to the Tokyo guidelines [2, 15] were excluded. Grade II AC is characterized by significant local inflammation, including gangrenous or emphysematous cholecystitis, pericholecystic abscess, or hepatic abscess. Grade III AC is defined as AC associated with target organ dysfunction, such as cardiovascular compromise (mean arterial pressure < 65 mm-Hg) requiring inotropic support, neurological impairment (e.g. decreased level of consciousness), or respiratory or renal dysfunction [15]. Patients with concurrent conditions such as gallstone pancreatitis, choledocholithiasis, malignancies of the hepatobiliary or pancreatic systems were also excluded.

PoCUS was performed at the physician’s discretion. PoCUS was included in emergency residency training at the NTUH. All residents and attending physicians were credentialed and approved to perform biliary PoCUS. They were evaluated by senior instructors and successfully passed a hands-on assessment, including biliary PoCUS. The instructors, certified by the Taiwan Society of Ultrasound in Medicine, had over 10 years of experience in performing sonographic examinations. All PoCUS examinations were documented in a standardized report format, which included the indication, sonographic findings, sonographic diagnosis, and management plan.

Surgeons were routinely consulted. Patients with AC may undergo emergency surgical intervention or non-operative management, which can include antibiotics alone or antibiotics in combination with percutaneous cholecystostomy (PTC).

### Data collection

Patients were divided into two groups: the PoCUS-first group and the CT-first group. The data were obtained from the electronic medical records, including age, sex, comorbidities, attending physician, clinical symptoms including right upper quadrant (RUQ) pain and fever (body temperature higher than 38.3 °C), laboratory data including white blood cell (WBC) counts and C-reactive protein (CRP), time of visits, door-to-physician time, door-to-US time, door-to-CT time, time to surgical consultation, door-to-PTC time, and ED-LOS, as well as the sonographic findings. The time of visits was categorized into weekday visits or weekend/holiday visits, as well as day-shift (8 a.m. to 6 p.m.) or night-shift visits (6 p.m. to 8 a.m.). ED-LOS was defined as the time interval from patient registration to leaving the ED (ward admission or emergency cholecystectomy). The time to surgical consultation was defined as the time interval from patient registration to the initiation of the surgical consultation, marked by the time the consultation form was submitted.

### Outcome measurement

The primary outcome was the relationship between the door-to-US/CT time and ED-LOS. The secondary outcome was the relationship between the door-to-US/CT time and time to surgical consultation.

### Statistical analysis

All data were analyzed by SAS software (SAS 9.4, Cary, North Carolina, USA). The Shapiro–Wilk test was conducted to assess the normality of continuous variables, including age, pain duration, WBC counts, CRP, door-to-physician time, door-to-US time, door-to-CT time, time to surgical consultation, door-to-PTC time, and ED-LOS. None of these variables followed a normal distribution (all *p* < 0.0001); therefore, they were reported as medians with interquartile ranges (IQRs) and analyzed using the Wilcoxon rank-sum test. Categorical data were expressed in counts and proportions and analyzed using a Chi-square test or Fisher exact test.

The linear regression model was used to examine the relationship between door-to-US time in the PoCUS-first group and ED-LOS. Covariates included age, sex, comorbidities, attending physician, clinical symptoms, laboratory data, time of visits, and door-to-physician time. If door-to-US time was found to be significantly associated with ED-LOS, it was further categorized into intervals of 60, 90, and 120 min to assess the impact of different timing thresholds.

Similarly, a linear regression model was employed to analyze the relationship between door-to-CT time in the CT-first group and ED-LOS. If a significant association was identified, door-to-CT time was also categorized into 60, 90, and 120-min intervals, following the same method.

Additionally, the relationship between door-to-US time in the PoCUS group and time to surgical consultation, as well as door-to-CT time in the CT-first group, was analyzed using the linear regression model and the same analytical approach. A p-value of less than 0.05 was considered statistically significant.

## Results

### Characteristics of study subjects

**Fig. 1:**
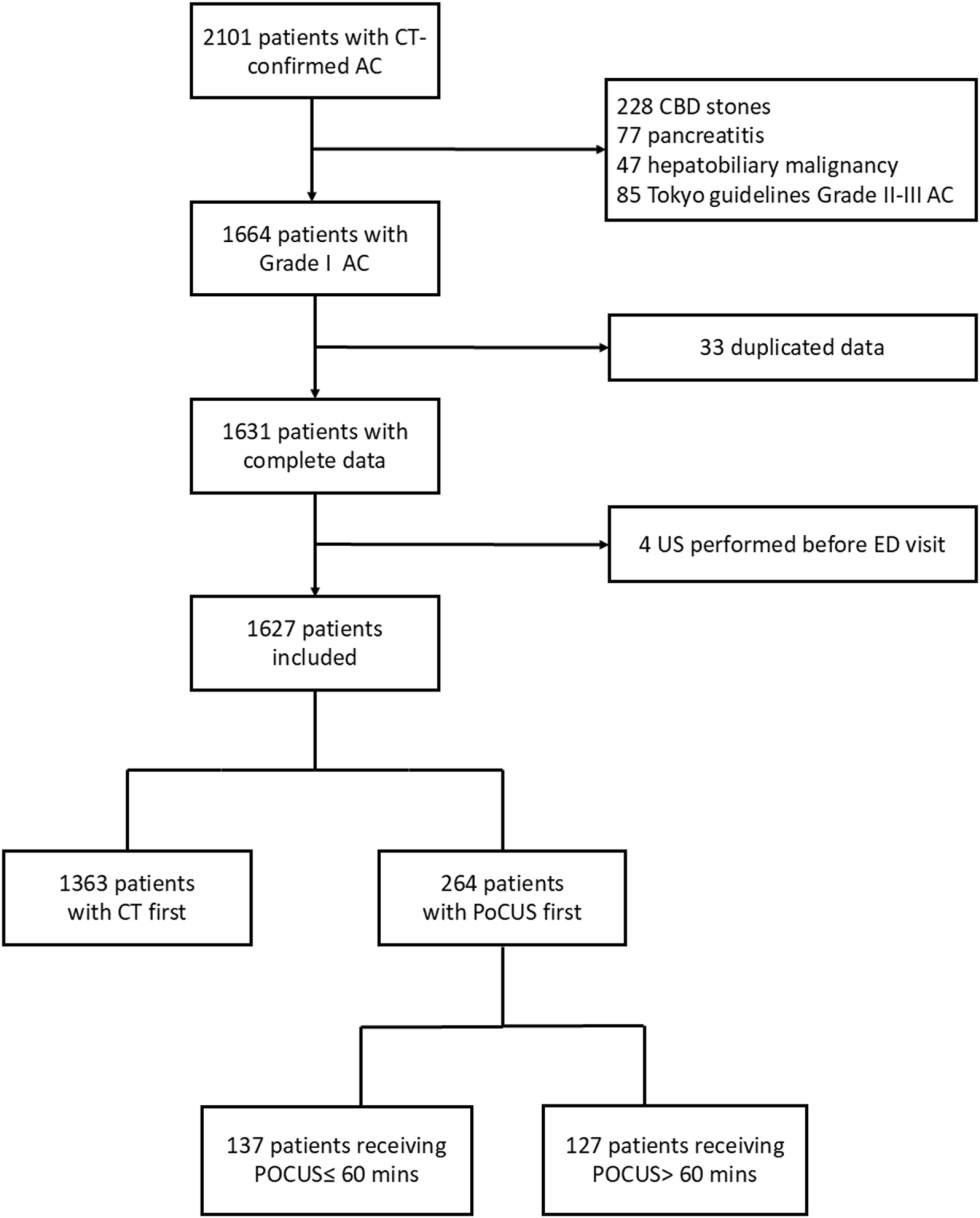
The study flowchart. CT, computed tomography; AC, acute cholecystitis; US, ultrasound; ED, emergency department; PoCUS, point-of-care ultrasound

During the study period, 2101 patients with CT-confirmed AC were eligible. After excluding 474 patients, 1627 patients were included in the analysis (Fig. 1).

PoCUS was the initial imaging modality for 264 patients (PoCUS first group, 16%), while the remaining patients underwent CT first (Table 1). Only one patient receiving CT first died in the ED. The characteristics of the 292 patients (18%) who underwent emergency cholecystectomy are detailed in Supplementary Table 1.

**Table 1:** The characteristics of the included patients

Variables	Total	CT first	PoCUS first	*p*-Value^‡^
	(n = 1627)	(n = 1363)	(n = 264)	
Age, years*	61 (47–73)	61 (48–74)	59 (45–71)	0.020
Male, n (%)	940 (58)	783 (57)	157 (60)	0.542
Comorbidity, n (%)				
Hypertension	592 (37)	506 (37)	86 (33)	0.153
Diabetes mellitus	391 (24)	338 (25)	53 (20)	0.099
Malignancy	229 (14)	203 (15)	26 (10)	0.031
Coronary artery disease	197 (12)	169 (12)	28 (11)	0.423
Chronic kidney disease	104 (6)	88 (7)	16 (6)	0.806
Cerebrovascular disease	112 (7)	97(7)	15 (6)	0.398
Congestive heart failure	64 (4)	57 (4)	7 (3)	0.24
RUQ pain, n (%)	987 (63)	821 (63)	166 (64)	0.582
Pain duration, days*	2 (1–3)	2 (1–3)	2 (1–3)	0.566
Fever, n (%)	463 (28)	411 (30)	52 (20)	< 0.001
Laboratory data*				
WBC, 10^3^/μL	11.36 (8.32–14.69)	11.32 (8.29–14.84)	11.40 (8.46–14.46)	0.798
CRP, mg/dL	5.56 (1.29–16.52)	6.08 (1.40–16.83)	3.17 (0.72–11.43)	0.128
Total bilirubin, mg/dL	0.95 (0.61–1.66)	0.96 (0.61–1.68)	0.94 (0.61–1.61)	0.078
Weekend/holiday visit, n (%)	459 (28)	388 (29)	71 (27)	0.603
Nightshift visit, n (%)	824 (51)	666 (49)	158 (60)	0.001
Door-to-physician time, mins*	19 (13–28)	19 (13–28)	20 (13–28)	0.908
Door-to-PoCUS^†^ time, mins*	167.5 (46–1061)	1063 (407–2056)^§^	55.5 (30–128)	< 0.001
Door-to-CT^†^ time, mins*	146 (94–258)	143 (93–240)	184.5 (106–459)	0.001
Time to surgical consultation, hrs*	7.1 (4.6–11.9)	6.9 (4.6–11.5)	7.7 (4.7–13.8)	0.108
Patients receiving PTC^†^, n (%)	478 (29)	410 (30)	68 (26)	0.154
Door-to-drainage time, hrs*	16.8 (8.9–26.0)	16.7 (8.8–24.8)	17.3 (9.7–33.2)	0.862
Emergency cholecystectomy, n (%)	292 (18)	246 (18)	46 (18)	0.817
ED Length of stay, hrs*	36.1 (15.3–64.3)	35.9 (15.3–64.3)	38.1 (15.3–64.8)	0.724

^*^Presented as median and interquartile ranges (IQRs)

^†^*RUQ* right upper quadrant; *WBC* white blood cell; *CRP* C-reactive protein; *PoCUS* point-of-care ultrasound; *CT* computed tomography; *PTC* percutaneous cholecystostomy; *ED* emergency department

^‡^Comparisons between patients receiving CT first or PoCUS first

^§^In CT first group, there were 364 patients receiving PoCUS after CT

The sonographic findings were displayed in Supplementary Table 2. The sensitivity of US in PoCUS first group was 83% (95% CI 78–87%).

#### LOS

The median LOS for all patients was 36.1 h. No significant differences in ED-LOS were observed between PoCUS first and CT first groups.

In the PoCUS first group, door-to-US time was positively associated with ED-LOS in the linear regression analysis (β = 0.27, *p* < 0.001). Other factors were unremarkable. Table 2 summarizes the impact of varying door-to-US time intervals, highlighting that conducting PoCUS within 60 min was associated with a reduced ED-LOS, resulting in a specific saving of 22.4 h, compared with those with PoCUS more than 60 min (Table 3).

**Table 2:** The effect of different timing of point-of-care ultrasound and computed tomography on length of stay and time to surgical consultation

	60 min	90 min	120 min
PoCUS-first group			
ED length of stay	− 0.203^†^ (*p* = 0.002)	− 0.220 (*p* = 0.001)	− 0.188 (*p* = 0.003)
Time to surgical consultation	− 0.281 (*p* < 0.001)	− 0.213 (*p* = 0.004)	− 0.204 (*p* = 0.007)
CT-first group			
ED length of stay	− 0.045 (*p* = 0.108)	− 0.045 (*p* = 0.107)	− 0.079 (*p* = 0.005)
Time to surgical consultation	− 0.067 (*p* = 0.038)	− 0.158 (*p* < 0.001)	− 0.199 (*p* < 0.001)

*PoCUS point-of-care ultrasound; *ED* emergency department; *CT* computed tomography

^†^Theβ-value in the linear regression model, after adjusting other covariates

**Table 3:** The length of stay and time to surgical consultation in the point-of-care ultrasound first group and computed-tomography first group

	PoCUS* first group			CT* first group		
	Door-to-US* time			Door-to-CT time		
	< 60 min	> 60 min	p-value	< 120 min	> 120 min	*p*-value
ED* length of stay, hrs^†^	26.9 (13.0–54.6)^‡^	49.3 (21.4–74.4)^§^	< 0.001	28.6 (11.8–50.9)^‡^	40.6 (17.5–69.5)^§^	< 0.001
Time to surgical consultation, hrs	5.7 (4.2–8.5)^a^	10.1 (6.7–21.0)^b^	< 0.001	5.5 (3.7–7.9)^a^	8.6 (5.6–13.5)^b^	< 0.001

^*^ED emergency department; *PoCUS* point-of-care ultrasound; *US* ultrasound; *CT* computed tomography

^†^Presented as median and interquartile ranges (IQRs)

^‡^p = 0.736

^§^p = 0.115.

^a^p = 0.119

^b^p = 0.004

In the CT-first group, door-to-CT time was also positively associated with ED-LOS in the linear regression analysis (β = 0.21, *p* < 0.001). No other significant factors were identified. Table 2 shows that performing CT within 120 min was associated with a reduced ED-LOS, resulting in a decrease of 12 h (Table 3). Additionally, 224 patients (16%) in the CT-first group underwent US following CT although no management changes occurred, with a median time from CT to US of 836 min (IQR, 253–1543 min). Patients who underwent CT alone had a shorter ED-LOS (Supplementary Table 3).

Further, the ED-LOS was similar between patients receiving PoCUS within 60 min in PoCUS first group and those receiving CT within 120 min in the CT first group (Table 3).

### Time to surgical consultation

There were no significant differences in time to surgical consultation between PoCUS first and CT first groups (Table 1).

In the PoCUS first group, door-to-US time was positively associated with surgeon arrival time in the linear regression analysis (β = 0.36, *p* < 0.001). Other factors were unremarkable. Table 2 indicates that performing PoCUS within 60 min was associated with a reduced time to surgical consultation with a specific saving of 266 min (Table 3).

In the CT-first group, door-to-CT time was also positively associated with time to surgical consultation in the linear regression analysis (β = 0.75, *p* < 0.001). No other significant factors were identified. Table 2 demonstrates that performing CT within 60, 90, or 120 min was associated with earlier surgeon arrival. Table 3 illustrates the results using the optimal CT timing (120 min), balancing its positive effects on both LOS and time to surgical consultation.

The time to surgical consultation was comparable between patients who received PoCUS within 60 min in the PoCUS-first group and those who underwent CT within 120 min in the CT-first group (Table 3).

## Discussion

Our study examines the impact of PoCUS/CT on ED-LOS and time to surgical consultation in patients with mild AC. Patients in the PoCUS-first and CT-first groups showed similar overall ED-LOS and time to surgical consultation. Notably, PoCUS performed within 60 min in the PoCUS-first group significantly reduced ED-LOS and facilitated earlier surgical consultation, as well as CT performed within 120 min in the CT-first group. Our findings suggest that the timely use of PoCUS and CT is associated with improved clinical outcomes, including a reduction in ED-LOS and expedited time to surgical consultation. This underscores the importance of performing PoCUS or CT at optimal times to enhance patient flow.

Modern EDs not only deliver urgent care for life-threatening conditions but also provide diagnostic assessments for a wide range of diseases [16]. Consequently, ED-LOS has significantly increased, leading to crowding and potential complications [17]. While the optimal time target for patients with AC remains uncertain, ED-LOS of less than 36 h has been frequently reported [10, 18, 19]. Furthermore, previous studies have shown that the time to surgical consultation in ED patients typically ranges from 3 to 12 h [10, 19]. In our study, the overall time to surgical consultation in the PoCUS group and the CT group fell within these reported benchmarks although the ED-LOS was higher.

While cholecystectomy remains the gold-standard treatment for AC, non-operative management has emerged as a proposed alternative in recent years [20, 21]. Our findings indicate that patients undergoing emergency cholecystectomy had a shorter ED-LOS, with a median time of approximately 15 h when following a streamlined workflow from ED presentation to operative unit transfer and subsequent ward admission. Reported median times from presentation to surgery in the literature range from 10 to 34 h [10–12], and our results align with this range. Notably, the proportion of patients undergoing emergency cholecystectomy (18%) was comparable between the CT-first and PoCUS-first groups, resulting in similar overall ED-LOS for both cohorts. The majority of patients in this study received non-operative management, which likely contributed to the longer ED-LOS observed compared to the durations reported in the literature. Furthermore, ED admissions, which accounted for less than 20% of total hospital admissions, were associated with a prolonged ED-LOS.

PoCUS is considered a “21st-century stethoscope” to evaluate a broad spectrum of illnesses and possibly change the management in emergency settings [22, 23]. PoCUS offers several advantages, including real-time imaging, non-invasiveness, and the absence of ionizing radiation. Unlike CT, PoCUS does not require waiting for renal function data, allowing for quicker diagnostic decisions. While previous studies primarily assessed the diagnostic accuracy of PoCUS for AC by comparing it with CT scans, operative notes, or pathological reports [24–26], few have explored its integration into clinical workflows. One notable study found that PoCUS performed by surgeons had a mean diagnostic time of 2.5 h, significantly shorter than the 11.9 h required for radiologist-performed US [24]. In our study, although the ED-LOS was similar between patients receiving CT within 120 min and those receiving US within 60 min, the diagnosis was made earlier in patients who underwent PoCUS first. This underscores the potential of PoCUS to expedite clinical diagnosis.

There are possible explanations for why PoCUS facilitates management process. PoCUS is performed at the bedside by the treating physician, eliminating the need to wait for radiology department availability or patient transport. The rapid diagnostic capability of PoCUS enables a more efficient clinical pathway, allowing ED physicians to quickly determine the need for collaboration with other specialties, thereby streamlining workflow and enhancing overall efficiency. Scholars have underscored the need to improve inter-clinician communication to enhance ED patient flow and increase patient safety [14, 27]. The real-time findings from PoCUS provide timely information and facilitate quicker and more direct communication and coordination with surgical or radiological teams, enabling faster management process. At our hospital, ED physicians promptly communicated with surgeons after performing PoCUS although formal surgical consultations were generally initiated only after CT confirmation.

Also, physicians who are willing to perform US may be relatively more proactive [28, 29], as this often indicates a tendency to actively seek immediate information to expedite decision-making. Moreover, the value of PoCUS is particularly significant during night shifts when radiology reports are not readily available. In our study, a higher proportion of patients presenting during night shifts underwent PoCUS as the initial diagnostic modality.

The characteristic sonographic findings of AC are thickened gallbladder wall, gallbladder distention, incarcerated gallstone, pericholecystic fluid collection, and sonographic Murphy’s sign [30, 31]. The distribution of sonographic findings was similar between PoCUS performed in under 60 min and over 60 min. The sensitivity observed in the PoCUS group was consistent with values reported in the literature [32, 33]. However, true-negative cases were not included, which prevented the evaluation of other diagnostic performance metrics for PoCUS.

This study had limitations. First, its retrospective design meant that certain clinical details, such as specific symptoms, patient flow, and physicians' discretion in selecting PoCUS or CT, were not documented. However, key symptoms of AC (RUQ pain and fever) were routinely recorded and included in the analysis. Additionally, door-to-physician time was accounted for, serving as a partial proxy for patient presentation time. Second, the data were collected from a single institution with active US training and ready access to US devices. This limits generalizability, and further validation in other emergency settings is necessary. Third, as a tertiary medical center, our hospital's patient population had more severe and complex comorbidities. A higher proportion of patients with malignancies received CT first in our study. Nevertheless, the effect of PoCUS on ED-LOS remained significant after adjusting for covariates, including comorbid conditions. Fourth, there was an imbalance in the number of patients between the PoCUS-first and CT-first groups, which may have introduced selection bias. This discrepancy reflects real-world practice, where ED physicians often have individual preferences for using US or CT during evaluations. Implementing a randomized study design could help eliminate this bias and provide a more accurate assessment of the clinical efficacy of PoCUS and CT. Fifth, no patients with mild AC at our hospital were managed using PoCUS alone. All patients with mild AC were included in the analysis. Sixth, we did not compare PoCUS performed by ED physicians with US conducted by radiologists, as all sonographic examinations for ED patients at our hospital were exclusively performed by ED physicians. Seventh, although ED physicians promptly communicated with surgeons after performing PoCUS, formal surgical consultations were generally initiated only after CT confirmation. This practice may have contributed to the similar time to surgical consultation observed between the PoCUS-first and CT-first groups. Eighth, we excluded patients with grade II and III AC, so our results may not be generalizable to those patient groups. Ninth, we collected data only up to June 2020 due to the onset of the COVID-19 pandemic. During the pandemic, ED visits declined significantly [34], and management processes were likely impacted by factors such as universal precautions, enhanced disinfection protocols, and other pandemic-related changes [35]. Tenth, although the ED-LOS for patients undergoing emergent cholecystectomy fell within the time frames reported in the literature [36], the overall ED-LOS was longer than expected [9]. A substantial percentage of patients received non-operative management, which may explain the extended ED-LOS and limit the generalizability of the findings. Also, our hospital is an academic medical center where ED admissions account for only 18% of total hospital admissions. A shortage of hospital beds significantly impacts patient flow, making the facilitation of ED processes a critical component of our mission to reduce ED crowding. Eleventh, this study did not evaluate whether the PoCUS-first group incurred lower overall medical costs. However, Taiwan's National Health Insurance (NHI) system, a compulsory single-payer social insurance program, provides coverage for approximately 99% of its 23 million residents [37]. Under this system, patients pay 10–30% of their medical expenses, with the remainder covered by the NHI. As a result, assessing medical costs in Taiwan is challenging to generalize for external comparisons. Finally, this study only included patients with CT-confirmed AC, excluding false-positive and true-negative cases. As a result, the diagnostic accuracy of PoCUS beyond sensitivity could not be fully assessed. Additionally, the impact of varying training levels on diagnostic accuracy was not evaluated. However, PoCUS performed by residents was supervised by attending physicians, likely minimizing the influence of training variability on the results.

## Conclusions

While PoCUS-first and CT-first approaches resulted in comparable ED-LOS and time to surgical consultation for patients with mild AC, performing PoCUS within the first 60 min or CT within 120 min was associated with shorter ED-LOS and earlier surgical consultation, enhancing the efficiency of ED management in patients with mild AC.

## Supplementary Information

Additional file 1.

Additional file 2.

Additional file 3.

## Data Availability

Data is provided within the manuscript and supplementary information files.

## Abbreviations

AC: Acute cholecystitis
ED: Emergency department
US: Ultrasound
CT: Computed tomography
PoCUS: Point-of-care ultrasound
LOS: Length of stay
NTUH: National Taiwan University Hospital
PTC: Percutaneous cholecystostomy
RUQ: Right upper quadrant
WBC: White blood cell
CRP: C-reactive protein
IQR: Interquartile range
NHI: National Health Insurance
