# Supplementary material for: Impact of a POCUS-first versus CT-first approach on emergency department length of stay and time to surgical consultation in patients with acute cholecystitis: a retrospective study
Source: Scand J Trauma Resusc Emerg Med. 2025 Feb 10;33:28. doi: 10.1186/s13049-025-01341-2 (PMC11812236; doi:10.1186/s13049-025-01341-2)
Supplement: Supplementary file 1 — Additional file 1. [file 13049_2025_1341_MOESM1_ESM.docx]

| **Supplementary Table 1. The comparison between patients receiving emergency cholecystectomy and non-operative management.** | | | |
| --- | --- | --- | --- |
| Characteristics | Emergency cholecystectomy | Non-operative management | p-Value |
|  | (n= 292) | (n=1335) |  |
| Age, years^*^ | 57 (41-67) | 62(49-74) | <0.001 |
| Male, n (%) | 169 (58%) | 771 (58%) | 0.969 |
| Right upper quadrant pain, n (%) | 230 (79%) | 757 (59.%) | <0.001 |
| Pain duration, days^*^ | 2 (1-2) | 2 (1-3) | 0.502 |
| Fever, n (%) | 61 (21%) | 402 (31%) | 0.001 |
| Weekend/holiday visit, n (%) | 77 (26%) | 382 (29%) | 0.440 |
| Nightshift visit, n (%) | 168 (58%) | 656 (49%) | 0.010 |
| ED^†^ Length of stay, hrs^*^ | 15.6 (9.9-23.0) | 43.5 (19.8-70.5) | <0.001 |
| ^*^presented as median and interquartile ranges (IQRs).  ^†^ED, emergency department. | | | |
