# Supplementary material for: Impact of a POCUS-first versus CT-first approach on emergency department length of stay and time to surgical consultation in patients with acute cholecystitis: a retrospective study
Source: Scand J Trauma Resusc Emerg Med. 2025 Feb 10;33:28. doi: 10.1186/s13049-025-01341-2 (PMC11812236; doi:10.1186/s13049-025-01341-2)
Supplement: Supplementary file 2 — Additional file 2. [file 13049_2025_1341_MOESM2_ESM.docx]

Supplementary Table 2. The sonographic findings of the included patients.

| Findings | PoCUS^*^≦60 mins  (n=137) | PoCUS>60 mins  (n=127) | p-Value |
| --- | --- | --- | --- |
| Gallstone, n (%) | 130 (95) | 116 (91) | 0.253 |
| Gall bladder wall thickening, n (%) | 112 (82) | 108 (85) | 0.474 |
| Sonographic Murphy’s sign, n (%) | 131 (96) | 122 (96) | 0.857 |

*PoCUS, point-of-care ultrasound.
