# Supplementary material for: Impact of a POCUS-first versus CT-first approach on emergency department length of stay and time to surgical consultation in patients with acute cholecystitis: a retrospective study
Source: Scand J Trauma Resusc Emerg Med. 2025 Feb 10;33:28. doi: 10.1186/s13049-025-01341-2 (PMC11812236; doi:10.1186/s13049-025-01341-2)
Supplement: Supplementary file 3 — Additional file 3. [file 13049_2025_1341_MOESM3_ESM.docx]

| **Supplementary Table 3. The comparison between patients receiving ultrasound or not in the CT-first group.** | | | |
| --- | --- | --- | --- |
| Characteristics | Without PoCUS^†^ | PoCUS | p-Value |
|  | (n= 1139) | (n= 224) |  |
| Age, years^*^ | 61 (47-74) | 63 (48-74) | 0.854 |
| Male, n (%) | 661 (58) | 122 (55) | 0.323 |
| Right upper quadrant pain, n (%) | 698 (64) | 123 (56) | 0.033 |
| Pain duration, days^*^ | 2 (1-3) | 2 (1-3) | 0.751 |
| Fever, n (%) | 348 (31) | 63 (29) | 0.435 |
| Weekend/holiday visit, n (%) | 314 (28) | 74(33) | 0.097 |
| Nightshift visit, n (%) | 561 (49) | 105 (47) | 0.507 |
| Time to surgical consultation, hrs^*^ | 6.7 (4.6-11.2) | 8.0 (4.7-13.3) | 0.006 |
| ED^†^ Length of stay, hrs^*^ | 30.7 (13.9-60.9) | 49.1(27.5-83.7) | <0.001 |
| ^*^presented as median and interquartile ranges (IQRs).  ^†^ED, emergency department; PoCUS, point-of-care ultrasound. | | | |
